# A13 MECHANISM OF INTESTINAL ORGANOID TRANSPLANTATION IN ENHANCING EPITHELIAL REGENERATION DURING EXPERIMENTAL NECROTIZING ENTEROCOLITIS

**DOI:** 10.1093/jcag/gwae059.013

**Published:** 2025-02-10

**Authors:** B Li, A Zito, Y Xiong, H Liang, H Zhu, A Pierro

**Affiliations:** The Hospital for Sick Children, Toronto, ON, Canada; The Hospital for Sick Children, Toronto, ON, Canada; The Hospital for Sick Children, Toronto, ON, Canada; The Hospital for Sick Children, Toronto, ON, Canada; The Hospital for Sick Children, Toronto, ON, Canada; The Hospital for Sick Children, Toronto, ON, Canada

## Abstract

NOT PUBLISHED AT AUTHOR’S REQUEST

Please acknowledge all funding agencies listed below

**Funding Agencies**: CIHR

